# Leveraging Heterogeneous Catalyst Design Principles for Volatile PFAS Destruction through the Thermal Decomposition of CF_4_

**DOI:** 10.1021/acsomega.5c07477

**Published:** 2025-10-28

**Authors:** Benjamin P. Williams, Reagan Elia, Happiness Mbando, Jeffrey D. Wilke, Florentino B. De la Cruz

**Affiliations:** † Department of Chemistry and Biochemistry, College of Arts and Sciences, 4127 University of North Florida, 1 UNF Drive, Jacksonville, Florida 32224, United States; ‡ Department of Civil Engineering, College of Computing, Engineering and Construction, University of North Florida, 1 UNF Dr., Jacksonville, Florida 32224, United States

## Abstract

The mineralization of per- and polyfluoroalkyl substances could be enhanced with the use of catalysts to circumvent the large energy inputs needed to reach the high temperatures theoretically required for destruction of CF_4_, the most thermally stable product of incomplete destruction of PFAS. In this review, we aim first to outline the state-of-the-art catalysts designed for CF_4_ breakdown over the past several years. Then, we seek to apply the principles of heterogeneous catalyst design to identify underexplored avenues for improving destruction efficiency. Our key takeaways are (1) catalyst surface structure is key, with Lewis acidity, crystal structure, and the presence of functional groups directly affecting performance; (2) lower temperature requirements can expand the parameter space for catalyst development, with materials beyond alumina currently underexplored; and (3) further testing under industrial conditions should be performed on those catalysts that have been shown to be most promising in controlled laboratory settings. Together, the ideas presented can inform future catalyst design, with an overall goal of efficient and cost-effective PFAS mineralization at industrially accessible temperatures.

## Graphical abstract

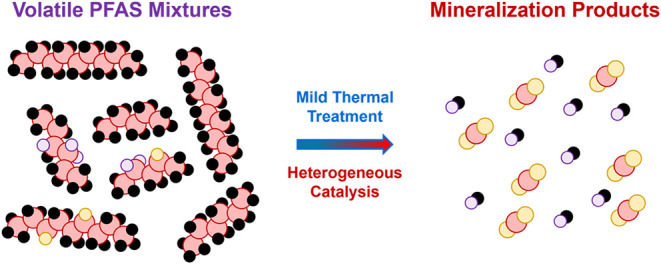

## 1 Introduction

There is growing concern with the wide proliferation of per- and polyfluoroalkyl substances (PFAS) due to their impact on both human health and the environment.^1,2^ To date, the US EPA has identified three key management strategies for PFAS: (1) thermal treatment, (2) landfilling, and (3) underground injection. Among these strategies, thermal treatment offers the potential advantage of mineralization, which prevents further release into the environment.^3^ However, current thermal treatment systems often yield products of incomplete destruction (PIDs) that can continue to have negative impacts.^4^ In the late 1990s, pioneering work at the National Institute of Standards and Technology investigated the stability of a wide range of (hydro)­fluorocarbons.^5^ Their work identified CF_4_ as particularly stable, which they attributed to the high strength (∼550 kJ mol^–1^) of the C—F bonds within the molecule. As CF_4_ has been identified as the stable end point of longer chain PFAS breakdown, effective CF_4_ mineralization could thus enable proposed multistep PFAS destruction processes (Figure 1).^6^ If employing active and stable catalysts, this two-step processing could be implemented in current thermal systems where temperatures do not reach those needed for uncatalyzed CF_4_ destruction.

**Figure 1:**
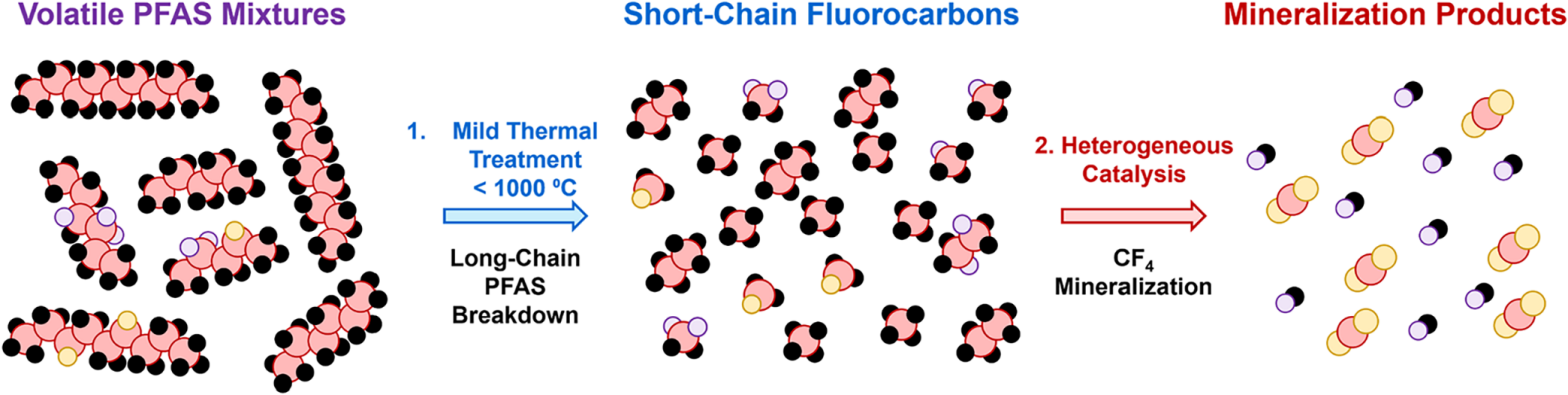
Proposed scheme for volatile PFAS mineralization. This review focuses on catalyst development for the most resilient product in Step 2, CF_4_. Carbon: red. Fluorine: black. Oxygen: yellow. Hydrogen: purple.

Theoretical estimation has found that a temperature of >1400 °C is required to mineralize CF_4_.^3,5^ However, laboratory, pilot, and full-scale studies have demonstrated that mineralization can be achieved at a lower temperature.^7^ Thermal mineralization of PFAS involves radical initiation, propagation, and branching. Linak and co-workers combined their kinetic model and Fourier-transform infrared (FTIR) data with experimental results collected from a Rainbow furnace.^8^ A detailed analysis of PIDs yielded destruction efficiencies (DEs) in agreement for CHF_3_ and C_2_F_6_, but overpredicted DEs for CF_4_ at high temperatures and underpredicted DEs for CF_4_ at low temperatures. In agreement with previous analysis, they identified the stability as CF_4_ > C_2_F_6_ > CHF_3_. Recent work by Weber et al. has further identified the generation of CF_4_ and C_2_F_6_ during the breakdown of perfluorooctanoic acid (PFOA) at temperatures >800 °C.^9^ Similar kinetic analysis for catalyzed CF_4_ destruction represents an area of opportunity for future studies.

The topic of catalytic thermal decomposition of CF_4_ has been reviewed previously.^10^ However, this Mini-Review will distinguish itself in two primary ways: (1) it will focus on recent (i.e., after 2021) literature, though earlier papers will be provided when appropriate for context; and (2) it will seek to further bridge the gap between PFAS destruction and heterogeneous catalyst design, suggesting areas where identified structure–function relationships may help to improve the efficiency of CF_4_ destruction.

## 2 State of Catalyst Development

The goal of this section is (1) to provide a historical context for the development of catalysts for CF_4_ destruction; (2) to illuminate the proposed mechanisms for CF_4_ destruction on the catalyst surface; and (3) to highlight recent work leveraging (1) and (2) to design new catalysts. Overall, this section will provide a foundation for the discussion presented in Section 3. Table 1 displays a summary table of catalyst composition, reported destruction efficiency, decomposition temperature, and operational stability.

**Table 1:** Summary Table of Catalysts for CF_4_ Destruction

composition	refs	reported DE (%)	operational *T* (°C)	operational stability
Ga-γ-Al_2_O_3_	^11^	>95	680	>18 h
Ce-AlPO_4_	^12^	>55	700	100 h
Ga/γ-Al_2_O_3_	^13^	98	630	>3 days
Ga/θ-Al_2_O_3_	^14^	100	600	>1000 h
γ-Al_2_O_3_	^15^	100	650	2 h
ZnAl_2_O_4_	^18^	>86	700	30 h
ZnAl_2_O_4_	^19^	>70	600	>160 h
Zn-θ-Al_2_O_3_	^20^	100	560	>250 h
Ce/γ-Al_2_O_3_	^21^	>45	650	50 h
Zr-γ-Al_2_O_3_	^22^	>78	650	60 h
Al_2_O_3_-ZrO_2_	^23^	100	580	>10 h
Hf-γ-Al_2_O_3_	^24^	100	650	>1000 min
Ni/ZrO_2_-γ-Al_2_O_3_ with WO_3_	^25^	>95	550	>30 h
Ni/γ-Al_2_O_3_	^26^	100	570	>300 h
MOR/CaO	^27^	>90	650	5 h
ZSM-5	^28^	80	500	<3 h
S/Ce/HZSM-5	^29^	>34	500	60 h

Over 20 years ago, El-Bahy et al. found that catalysts of 5% metal oxides on alumina were capable of inducing CF_4_ hydrolysis in the 580–680 °C temperature range.^11^ FTIR spectroscopy analysis found Lewis acid sites to be critical to activity, with Ga, Zn, and Zr oxides imparting the best activity. Ni, Cr, and Sn oxides also increase performance relative to bare γ-Al_2_O_3_. Ichikawa and his group later built on this study to identify 20% Ga/γ-Al_2_O_3_ as 15x more active than the 10% Ce-AlPO_4_ catalyst identified by Takita^12^ (see below).^13^ More recently, work by Cortés, Liu, and co-workers clarified the mechanism of CF_4_ destruction over Ga/θ-Al_2_O_3_.^14^ A combination of magic-angle spinning nuclear magnetic resonance spectroscopy (MAS NMR), in situ diffuse reflectance infrared Fourier transform spectroscopy (DRIFTS), and density function theory (DFT) found that Ga­(IV) reacts with H_2_O vapor to form Ga–OH, which assists with defluorination of poisoned Al active sites during the reaction.

More recent work has shed further light on the mechanism of CF_4_ destruction over alumina. Liu and co-workers used DFT calculations to identify that the Al­(III) sites on the (110) crystal plane of γ-Al_2_O_3_ strongly interact with CF_4_.^15^ Hydrothermal synthesis yielded γ-Al_2_O_3_ “nano-slices” that preferentially exposed the (110) plane. ^27^Al magic angle spinning NMR and in situ FTIR corroborated exposed Al­(III) as the active site. Liu, Coote, and co-workers built on this work by combining molecular dynamics (MD) simulations with synchrotron vacuum ultraviolet photoionization mass spectrometry.^16^ Here, they identified surface hydroxyl groups as key to breaking C—F bonds (Figure 2). Surface hydroxyl groups are also identified to repopulate oxygen vacancies generated during catalysis, with this “surface healing” process proposed to occur through the Mars-Van Krevelen mechanism. They notably found CF_2_O and CO in addition to CO_2_. Oh et al. have suggested that CF_4_ hydrolysis proceeds in two steps, with dissociative adsorption of CF_4_ at a Lewis acid site forming CF_2_O and then proceeding to CO_2_.^17^ Since C–F bond breaking is rate limiting, many routes may thus be open to CF_2_O formation. HF detachment is also difficult, which may induce catalyst deactivation. Jeon et al. found that Zn promotes catalysis on γ-Al_2_O_3_.^18^ Specifically, a molar ratio of Zn:Al of 0.1 generated ZnAl_2_O_4_ that preferentially adsorbed CF_4_ over H_2_O vapor at Lewis acid sites and prevented the detrimental γ-Al_2_O_3_ to α-Al_2_O_3_ phase transition. A similar understanding led Liu and co-workers to treat ZnAl_2_O_4_ with H_2_SO_4_ to increase acidic Al­(III) sites and yield better performance.^19^ More recently, Luo et al. built on this work by modifying θ-Al_2_O_3_ with Zn to generate Lewis and Brønsted acid pairs that showed effective CF_4_ conversion at 560 °C for more than 250 h.^20^ Song et al. likewise found an enhancement mechanism for Ce/γ-Al_2_O_3_ modified by cerium sulfate.^21^ Modification with Ce made the alumina more resistant to transformation to AlF_3_ and active Lewis acid site density was increased by sulfate.

**Figure 2:**
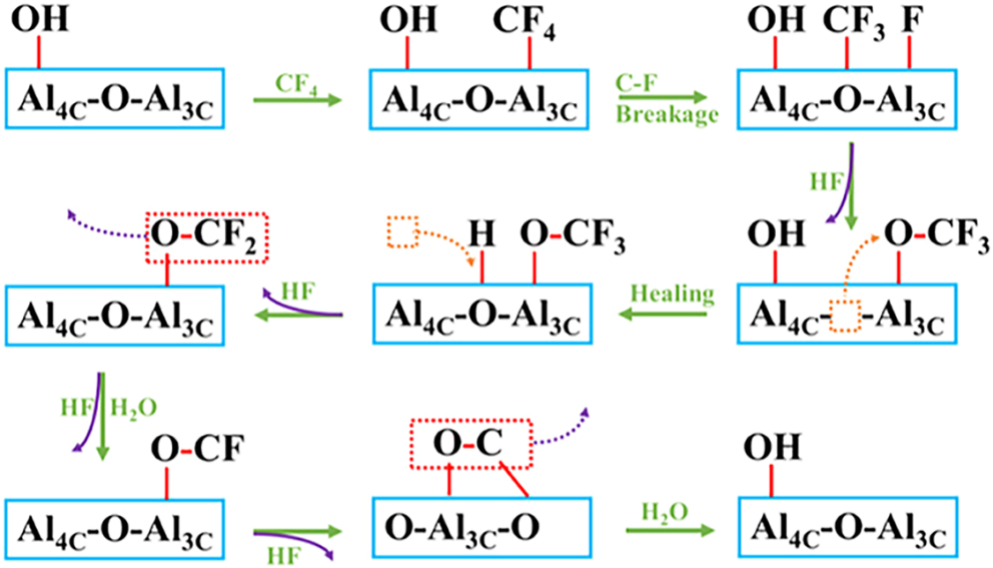
Experimentally corroborated MD simulations illuminate a pathway for CF_4_ destruction on alumina. Modified with permission from ref 16.

Shen, Liu, and co-workers found that Zr could also improve the activity of γ-Al_2_O_3_ for CF_4_ destruction.^22^ 16% Zr on γ-Al_2_O_3_ showed the best performance, with 85% conversion at 650 °C, remaining relatively stable at 78% conversion after 60 h. NH_3_-temperature-programmed desorption (TPD), FTIR, and X-ray photoelectron spectroscopy (XPS) analysis suggest electron transfer from Zr to Al increases the Lewis acidity of the surface. Liu and co-workers applied sulfated Al_2_O_3_ dispersed on a ZrO_2_ nanosheet to achieve a reported CF_4_ DE of 100% at 580 °C.^23^ DFT, IR, and XPS analysis suggests that protonated sulfate species (-HSO_4_) can activate the C–F bond in adsorbed CF_4_. Liu and co-workers further leveraged density functional theory (DFT) calculations for rational catalyst design that capitalized on antibonding-orbital hybridization to activate the C–F bond.^24^ Hf-γ-Al_2_O_3_ showed especially strong performance, with reported 100% conversion at 650 °C. Jang et al. used a sol–gel synthesis to generate Ni/ZrO_2_-γ-Al_2_O_3_ catalysts modified with WO_3_ that decomposed CF_4_ with >99% efficiency at 550 °C.^25^ They attribute the increased catalytic activity to the ability of Ni and W to increase surface acidity, while ZrO_2_ improved stability by preventing Al_2_O_3_ sintering. Notably, they identified the interaction between CF_4_ and H_2_O vapor as playing a key role in catalysis. Liu and co-workers also found that Ni can improve the stability of Al_2_O_3_ catalysts by binding the adsorbed *COO species that can poison the catalytic surface during low temperature CF_4_ breakdown.^26^ The 10% Ni/γ-Al_2_O_3_ catalyst achieved reported 100% CF_4_ conversion at 570 °C for 300 h.

In addition to modified alumina, zeolites can provide favorable surface structure for CF_4_ destruction. Araki et al. showed that active zeolites can be combined with stable CaO to improve performance. Activity of modified CaO decreased as mordenite > ZSM-5 > β > Y, which followed the same trend as the surface acidity determined by NH_3_-TPD.^27^ Mdlovu et al. identified an analogous trend, ZSM-5 > β > Y, and performed a detailed kinetic analysis to find a rate constant of 1.96 × 10^–5^ min^–1^ for CF_4_ decomposition over ZSM-5.^28^ Zheng et al. used both Ce and H_2_SO_4_ to increase acid sites in HZSM-5, imparting the inactive native HZSM-5 with a 41% conversion efficiency for CF_4_ conversion at 500 °C.^29^ NH_3_-TPD analysis found that the acid treated catalyst (S/Ce/HZSM-5) possessed substantially more strong acid sites than the bare (HSZM-5) or Ce-modified (Ce/HZSM-5) catalyst, leading to significantly higher activity (Figure 3).

**Figure 3:**
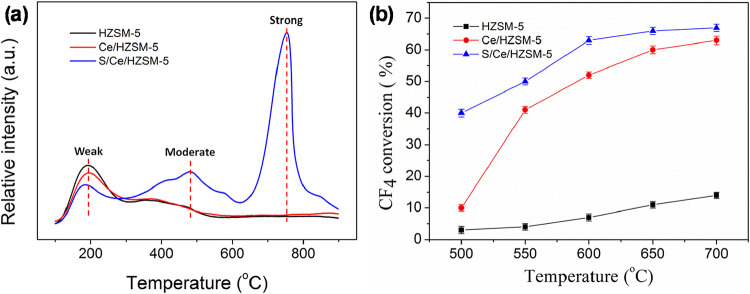
(a) NH_3_-TPD shows evidence of increased moderate and strong populations of acid sites on the S/Ce/HZSM-5 surface. (b) S/Ce/HZSM-5 shows a commensurate increase in activity for CF_4_ conversion. Modified with permission from ref 29.

## 3 Future Outlook

A few key points can be highlighted from the work presented above. The Lewis acidity of the surface is paramount to imparting high CF_4_ DEs, with both the strength and prevalence of acidic sites affecting performance. Other aspects of the surface can also play a role, whether by stabilizing the parent crystal structure, providing additional functional groups as a mechanism for surface healing, or assisting in the catalytic cycle. Effective future catalyst design should take these features into account, including by leveraging computational analysis for rational design.^24^

The success of zeolites suggests that using the understanding generated in alumina-based catalysts to expand the library of catalyst materials could push performance to higher levels. Efficient catalysts that can lower the temperature needed for CF_4_ destruction allow for an expansion of the parameter space that can be explored in catalyst design. Zeolite supports provide additional tunability not found with Al_2_O_3_, including greater porosity and an increased density of acidic sites. Zeolites can incorporate a wide variety of secondary metals, which can improve catalytic activity and stability. To identify the most effective secondary metals, more fundamental work is needed to elucidate the interactions between metal centers and adsorbed species that drive the catalytic process. Here, we define fundamental work as the rational modification of catalysts in a controlled lab setting, with a goal of generating design rules that can be applied in industrial settings (which are discussed below). DRIFTS analysis, as presented by Liu and co-workers (Figure 4),^26^ is one promising route to a better understanding.

**Figure 4:**
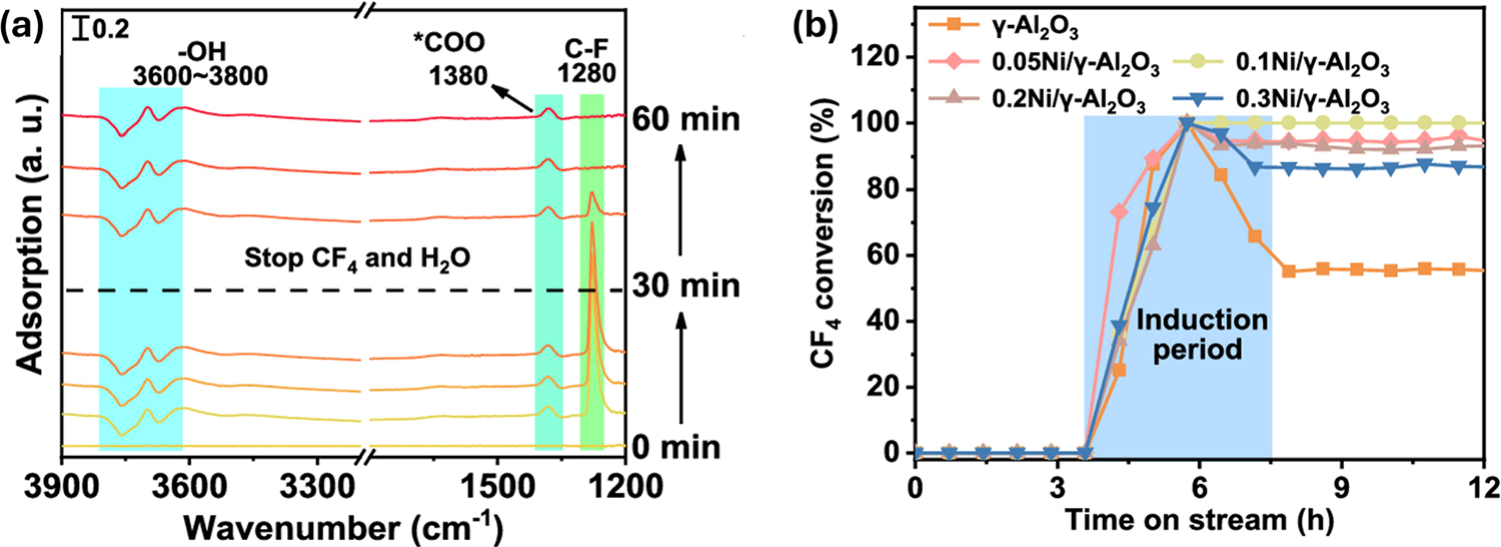
(a) DRIFTS analysis reveals a resilient *COO species bound to the catalyst surface. (b) The introduction of Ni is proposed to disrupt *COO binding to active sites resulting in stable conversion for 10% Ni/γ-Al_2_O_3_. Modified with permission from ref 26.

Along with tunable supports, the catalytic surface itself can be better tailored to CF_4_ destruction. One framework for designing heterogeneous nanoparticle catalysts is to tune the metal d-band center for ideal adsorption strength, as dictated by the Sabatier principle. More computational work is needed to directly interrogate the ability of electronic surface structure to be optimized for ideal CF_4_ adsorption; that is, strong enough for reactions to take place but weak enough to prevent surface poisoning. Supports also play a critical role in determining catalyst electronic structure. Here again, the tunability allowed by lower temperature reactions could allow DEs to reach new heights.

Promising results generated in lab settings should further be confirmed under industrial conditions. The lower temperatures needed for catalytic conversion could allow for incorporation into existing engineering systems with minimal retrofitting. Catalyst stability and resistance to deactivation in systems with realistic gas streams should also be further explored. In addition to emissions from manufacturing plants and landfills, the management of treatment residuals from aqueous PFAS remediation, e.g., granular activated carbon (GAC), releases volatile PFAS.^2^ As an example application to address this issue, a catalytic thermal oxidizer unit may be installed downstream of the GAC reactivation process to further enhance PFAS mineralization. Incorporation into processes like these could allow heterogeneous catalysts to form the foundation for efficient and sustainable PFAS destruction.

## 4 Conclusions

Overall, the rational design of heterogeneous catalysts that can destroy CF_4_ at low temperatures holds great promise for PFAS mitigation. Work in recent years has identified the importance of surface structure in moving toward near-complete mineralization. As catalysts improve, the lower required temperatures enable the introduction of materials beyond alumina that could not survive the harsh conditions previously needed. Activity and stability at lower temperatures could further allow for straightforward modification of current infrastructure, e.g., retrofitting an existing thermal flare, that can drive PFAS destruction without incurring prohibitive additional costs. To build on current understanding, more work is needed to rationally design catalysts, guided by computational study, and to apply identified catalysts in unoptimized industrial conditions. Finally, while our current focus is on the mineralization of CF_4_ generated from thermal destruction of PFAS, the application of CF_4_ destruction technologies can be extended to other industrial sources, such as aluminum production and semiconductor manufacturing, which respectively accounted for 0.1 and 247.2 kt of CF_4_ in 2022.^30^
